# Oral health–related quality of life after early extraction of primary second molars in children with congenitally missing second premolars: a randomized controlled trial with matched controls

**DOI:** 10.1093/ejo/cjag048

**Published:** 2026-06-11

**Authors:** Julia Naoumova, Shaker Nawaia, Larisa Krekmanova

**Affiliations:** Department of Orthodontics, Institute of Odontology, Sahlgrenska Academy, University of Gothenburg, Box 450, 405 30 Gothenburg, Sweden; Public Dental Service, University Clinic of Orthodontics, 413 90 Gothenburg, Region Västra Götaland, Sweden; Public Dental Service, Clinic of Orthodontics, 541 30 Skövde, Region Västra Götaland, Sweden; Department of Paediatric Dentistry, Institute of Odontology, Sahlgrenska Academy, University of Gothenburg, Box 450, 405 30 Gothenburg, Sweden

**Keywords:** agenesis, hemisection, interceptive orthodontics, long-term follow-up, patient reported outcome

## Abstract

**Background:**

Extraction of mandibular primary second molars in patients with congenitally missing second premolars may influence oral health–related quality of life (OHRQoL), yet this has not been previously investigated.

**Objective:**

To assess the impact of mandibular primary second molar extraction on OHRQoL compared with an age- and gender-matched control group without dental agenesis.

**Design:**

Prospective, randomized, longitudinal split-mouth study.

**Methods:**

Patients were recruited from nine public dental clinics in the Skaraborg region, Sweden (2017–2020) with bilateral congenitally missing second mandibular premolars. Each mandibular side was randomly assigned to extraction or hemisection. At the 4-year follow-up, the intervention group underwent a clinical examination and completed the Child Perceptions Questionnaire (CPQ_11–14_) along with a treatment recall and perception questionnaire, whereas the control group underwent a clinical examination and completed the CPQ_11–14_.

**Results:**

The study included 80 patients, divided equally between the intervention group (*n* = 40) and the control group (*n* = 40). There was no significant difference in total CPQ scores between the intervention group (mean 8.5, SD 5.6) and the control group (mean 6.9, SD 5.6; *P* = 0.218), nor were any differences observed between sexes. The intervention group scored significantly higher in emotional (*P* = 0.018) and social well-being (*P* = 0.045). Hemisection was recalled as more painful than conventional extraction (*P* < 0.001). Despite 65% reported awareness of residual spaces, 82.5% were not interested in further treatment.

**Conclusion:**

Extraction of mandibular primary second molars in patients with congenitally missing second premolars did not adversely affect overall OHRQoL 4 years post-treatment. Hemisection was recalled as more painful than conventional extraction. Most patients were satisfied without additional treatment, indicating good long-term acceptance of residual spaces following interceptive extraction.

**Trial registration:**

The trial was registered with https://www.researchweb.org/is/sverige, registration number: 967125.

## Introduction

Agenesis, the congenital absence of teeth, is a common dental condition, caused by early developmental disruptions during tooth formation [1]. In Scandinavia, the second mandibular premolar is the most commonly missing tooth, with an occurrence rate of 3% to 3.3% in the population [2–5]. Of these cases, ∼43.5% to 47.7% is bilateral [6].

Management of second mandibular premolar agenesis includes several treatment approaches. One option is to retain the primary second molars, while another involves extracting them, either entirely or through hemisection, before eruption of the second permanent molars to promote spontaneous space closure. Hemisection of the primary second molar, with staged removal of the mesial and distal segments, is thought to promote more controlled spontaneous closure of the second premolar space compared with conventional extraction by facilitating parallel mesial movement of the first permanent molar, minimizing tipping, and preserving alveolar bone. In cases requiring orthodontic intervention, extraction may be followed by space closure using fixed appliances supported by Class II elastics or temporary anchorage devices (TADs). Less frequently used alternatives include autotransplantation of third molars into the premolar site, placement of dental implants, or fixed prosthetic restorations [7–11].

The impact of these procedures on children can be better understood through the concept of oral health-related quality of life (OHRQoL). According to Locker [12], OHRQoL reflects the extent to which oral disorders affect daily functioning in meaningful ways—impacts significant in severity, frequency, or duration to influence an individual's overall life experience. Similarly, Kressin [13] defined OHRQoL as encompassing not only traditional medical views of health but also how individuals perceive their oral health as influencing their well-being and ability to function in everyday life. In children, OHRQoL is often assessed through parental reports, as concerns have been raised regarding the reliability of children's self-assessments due to their limited cognitive and communication skills [14]. A recent systematic review [15] concluded that malocclusions negatively affect the OHRQoL of children and adolescents, particularly when the malocclusion involves the esthetic zone. As a self-perceived measure, OHRQoL is influenced by cultural and social contexts, leading to variations across countries and populations [16].

When comparing orthodontic treatments with and without premolar extractions, one study using the OHIP-14 questionnaire found that both groups experienced a temporary decline in OHRQoL after treatment initiation. However, the extraction group reported a greater and more prolonged decrease, especially in domains related to physical pain and social well-being. Recovery occurred within 3 months for the nonextraction group and within 6 months for the extraction group [17]. Previous research has also shown that children with a clear orthodontic treatment need reported higher levels of loneliness and poorer OHRQoL. Notably, a significant association between malocclusion and loneliness was observed in girls, moderated by self-esteem, whereas no such relationship was found in boys [18]. Tooth agenesis has been linked to both esthetic and functional difficulties, which may negatively influence children's OHRQoL [19, 20]. Despite this, limited research has focused on how different treatment strategies for malocclusion—particularly those addressing tooth agenesis—affect OHRQoL in younger children.

To address this gap, the present study aims to evaluate the impact of extracting mandibular primary second molars in children with congenitally missing second premolars and to compare the outcomes with those of an age- and gender-matched control group without dental agenesis.

## Subjects and methods

The study was conducted in accordance with the Declaration of Helsinki, and ethical approval was granted by the Regional Ethical Review Board at the University of Gothenburg (reg. no. 558-17). Written and verbal information about the study was provided to both the patients and their parents, and consent was obtained before participation. The first and second part of the study assessing space closure and dentoalveolar changes were published in 2025 [8 and 21].

### Trial design

The study was designed as a single-center, prospective, split-mouth randomized controlled clinical trial.

### Participants, setting, and eligibility criteria

Between 2017 and 2020, participants were recruited from nine public dental clinics in the Skaraborg region. The inclusion criteria were:

- Age 9–12 years.
- Bilateral agenesis of the mandibular second premolars.
- Bilateral persisting deciduous second mandibular molars.
- Bilateral presence and unerupted mandibular second permanent molars.

The exclusion criteria were:

- Angle class II: 2 (as mandibular extractions could potentially deepen the bite).
- Generalized spacing in the mandible (defined as ≥8 mm of spacing measured from the mesial right primary second molar to the mesial left primary second molar).
- Cleft lip and palate or other craniofacial deformities.

### Sample size

The sample size was determined for the initial phase of the trial, designed to detect a clinically meaningful difference of 2 mm (±1 mm) in space closure between the two extraction methods [8]. Assuming a type I error rate of 5% and a type II error rate of 10%, the required sample size was calculated to be 30 patients. A dropout rate of 30% was anticipated.

### Randomization

Patients were randomly allocated to receive either extraction or hemisection on the left or right side of the mandible. An independent author generated the randomization sequence using an online platform (https://www.graphpad.com/quickcalcs/randomize1.cfm) and ensured allocation concealment by using sealed, numbered envelopes prepared in advance [8].

### Intervention group

Interceptive extractions were carried out at the Orthodontic Clinic in Skövde, Sweden.

#### Hemisection

The deciduous tooth was divided at the furcation, and only the distal half was extracted, leaving the mesial half with exposed pulp untreated. Patients were examined every 8 weeks, and when the space between the first permanent molar and the remaining tooth decreased to < 2 mm, the mesial half was extracted using the same procedure.

#### Conventional extraction

The entire primary tooth was removed with forceps. Both procedures were performed on each patient with a 10–14-day interval between them to avoid overlapping postoperative symptoms. No participant received orthodontic treatment between baseline (T1) and follow-up (T2). The mean follow-up time was 4.2 (SD: 0.6) years. A detailed description of the intervention has been previously reported [8].

### Control group

The inclusion criteria for the control group consisted of children, with matched number of boys and girls, aged between 9 and 12 years. These children had no history of dental agenesis and displayed a typical dental development pattern for their age group, and they were selected from the same public Dental Clinics in Skövde, Sweden as the intervention group.

### Questionnaire

At T2, all patients in both the intervention and control groups completed the Swedish version of the validated short-form CPQ_11–14_  *questionnaire* [22], underwent a *clinical examination*, and participated in a *semi-structured interview*.

The CPQ_11–14_, widely used in dental research [10, 21–23], assesses the impact of OHRQoL of children aged 11–14 years. It consists of 16 questions covering four domains: OS- oral symptoms (pain, discomfort, or sensitivity), FL- functional limitations (effects on eating, speaking, or smiling), EW- emotional well-being (feelings of self-consciousness or embarrassment), and SW- social well-being (effects on interactions and participation in social activities). Each item is scored on a 5-point frequency scale from 0 (never) to 4 (every day), yielding a total score range of 0–64 and domain scores from 0 to 16. Higher CPQ_11–14_ scores indicate a lower OHRQoL, and a severe impact on OHRQoL was defined as one or more items scoring 3 or 4. In addition, two global self-rating questions with 5- point scale were added: measuring oral health with a range from excellent to poor and measuring overall well-being with a range from not effected at all to very much affected.

The clinical examination was performed by one of the authors (SN) and included assessment of malocclusion in the sagittal (CL I, CL II, CL III), vertical (open bite: <0 mm, deep bite: >2/3 of the mandibular incisors covered by maxillary incisors, normal bite: 0–2/3 coverage), and transversal (normal, cross-bite, scissors-bite) relationship, evaluation of enamel defects (hypoplasia, opacities, or absence of defects), and scoring using the IOTN-DHC. This index divides the treatment need into five different levels: (i) no need; (ii) mild/little need; (iii) moderate/borderline need; (iv) severe need; and (v) extreme treatment need [24]. Data on manifest caries in permanent teeth, recorded as decayed, missing, and filled surfaces (DMFS), were obtained from the most recent dental records, where a manifest caries lesion was defined as caries detected by probing or visible into dentine on bite-wing radiographs at the most recent regular recall visit to the public dental clinic.

The semi-structured interview addressed the history of trauma and headache frequency, with responses for headache frequency categorized as ‘No’ (never/a few times per year) or ‘Yes’ (monthly/weekly/daily). Additional questions were directed to the guardian, including educational level and current employment status. A low socio-economic level was defined as having at least one unemployed parent (excluding parents staying at home to look after children) and/or parents whose highest level of education was primary school (9 years of schooling). Patients were also asked whether they live with both parents. These factors were considered due to their potential influence on the child's overall health and quality of life.

The intervention group completed additionally two questionnaires: the *treatment recall and perception questionnaire* (TRPQ) and the *Children's Fear Survey Schedule – Dental Subscale* (CFSS-DS). The TRPQ is a six-item questionnaire with multiple-choice and VAS-scale responses which was developed to assess patients’ recollection of pain during the hemisection and extraction procedures, understanding of the treatment purpose, and their perception of the residual spaces. The CFSS-DS is a 15-item questionnaire assessing dental anxiety in children by evaluating their fear level related to dental visits and procedures. Each item is scored from 1 (‘not at all afraid’) to 5 (‘very afraid’), giving a total score range of 15–75. A score of 38 or higher indicates clinically significant dental fear. The CFSS-DS has been widely used and validated for internal consistency, reliability, and validity in various populations, including in Sweden [25].

### Blinding

This study followed a single-blind design, in which all data collection was carried out by an independent examiner who was blinded to the participants’ treatment assignments.

## Statistics

The data were statistically analysed using IBM SPSS Statistics (version 29.0; IBM Corp., Armonk, NY, USA). Chi-square analysis and Fisher’s exact test was used to assess categorical data. Independent T-tests and paired sample T-tests were used to compare mean scores, while Mann–Whitney U test was used to compare median scores. A multiple linear regression analysis was performed to assess whether IOTN-DHC affected the total CPQ_11–14_ score. The dependent variable was total CPQ_11–14_ score and IOTN-DHC as the independent discriminant, with possible confounders (sagittal relationship, vertical relationship, transversal relationship, caries, enamel defects, dental trauma, headache, co-habitation and socioeconomic level) systematically entered. A *P*-value of < 0.05 was considered statistically significant.

## Results

### Sample characteristics

**Figure 1:**
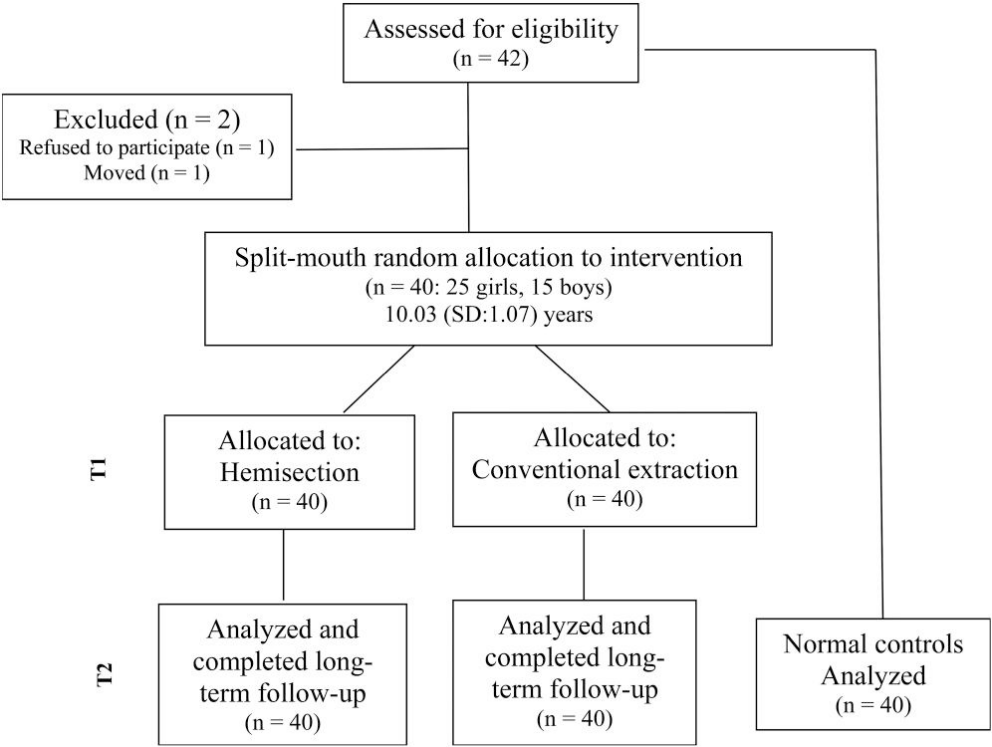
Flowchart illustrating the intervention and the control group.

The study included 80 patients, evenly divided between the intervention and control groups. Each group comprised 25 boys and 15 girls. The mean age at T2 in the intervention group was 14.72 years (SD = 1.25) for boys and 14.2 years (SD = 0.96) for girls, compared with 14.52 years (SD = 1.62) and 14.65 years (SD = 0.8), respectively, in the control group (Fig. 1).

Descriptive characteristics, including malocclusion, IOTN-DHC, caries, enamel defects, dental trauma or headache history, and socio-economic indicators (potential confounders), are shown in Table 1. The only significant differences seen between the intervention and control group was the IOTN-DHC (*P* = 0.001).

**Table 1:** Possible confounders in the intervention and the control group.

Confounders, *n* (%)	Intervention group (*n* = 40/%)	Control group (*n* = 40/%)	Group differences (*P*-value)
**Sagittal relationship**			
CL I	30/75	35/87.5	0.234
CL II	10/25	5/12.5	
CL III	0	0	
**Vertical relationship**			
Normal	32/80	34/85	0.152
Anterior open bite	0	0	
Deep bite	8/20	6/15	
**Transversal relationship**			
Normal relation	38/95	37/92.5	0.196
Cross-bite	1/2.5	3/7.5	
Scissors-bite	1/2.5	0	
**IOTN-DHC**			
No need	1/2.5	14/35	**0**.**001**
Mild/little need	15/37.5	8/20	
Moderate/borderline need	0	8/20	
Severe need	21/52.5	9/22.5	
Extreme treatment need	3/7.5	1/2.5	
**Caries (DMFS)**			
0	25/62.5	27/67.5	0.807
1	4/10	5/12.5	
2	6/15	3/7.5	
> 3	5/12.5	5/12.5	
**Enamel defects**			
Absence of defects	26/65	28/70)	0.456
Opacities	8/20	10/25	
Hypoplasia	5/12.5	2/5	
**Dental trauma**			
No	33/82.5	28/70	0.275
Yes	7/17.5	12/30	
**Headache**			
No	26/65	34/85	0.062
Yes	14/35	6/15	
**Co-habitation**			
Living with both parents	28/70	33/82.5	0.322
Not living with both parents	12/30	7/17.5	
**Low socioeconomic level**			
Yes	2/5	0	0.744
No	38/95	40/100	

Bold indicate *P* < 0.05.

### Treatment recall and perception

Overall, 75% (*n* = 30) of patients remembered the rationale for extraction of the primary second molars, and 60% (*n* = 24) correctly recalled the extraction strategy for each side of the mandible. When asked whether they experienced any difference between hemisection versus conventional extraction, 18 patients (45%) reported that hemisection was more painful, one patient (2.5%) reported greater pain during conventional extraction, and 21 patients (52.5%) reported no difference. Patients recalled mean pain score during hemisection as 4.3 (SD = 2.6; 95% CI: 3.5–5.2), compared with 3.7 (SD = 2.2; 95% CI: 3.0–4.5) during conventional extraction, with the difference being statistically significant (*P* < 0.001). The mean CFSS-DS score was 20.4 (SD 5.3), with no significant gender differences (*P* = 0.139).

Regarding awareness of the residual spaces, 26 patients (65%) reported thinking about the gaps, 11 (27.5%) did not, and three patients (7.5%) reported that the question was not applicable. Despite this, most patients (82.5%) did not wish to have any treatment for the remaining gap.

### Oral health-related quality-of-life

For the global rating of oral health, 90% of patients in the intervention group reported their health regarding the teeth, lips, jaws, and mouth as good, very good, or excellent, compared with 92.5% in the control group (*P* = 0.380). No gender differences were observed between the groups (*P* = 0.221). In terms of overall well-being, 90% of patients in the intervention group and 95% in the control group indicated that their oral health affected their lives not at all, occasionally and sometimes (*P* = 0.107). No significant gender differences were identified between the groups (*P* = 0.352).

No difference was observed in the mean total CPQ_11–14_ score between the intervention group (mean 8.5, SD 5.6) and the control group (mean 6.9, SD 5.6; *P* = 0.218) (Table 2). Similarly, no significant differences in total CPQ_11–14_ scores were found between girls and boys either in the intervention group (*P* = 0.134) or the control group (*P* = 0.736).The ranking of domains from highest to lowest score was OS, EW, SW, and FL in the intervention group, compared with OS, FL, SW, and EW in the control group (Table 2).

**Table 2:** Mean (SD) and median (range) score of CPQ_11–14_ (range 0–64), each domain (range 0–16) between the intervention and the control group.

	Intervention group (*n* = 40)		Control group (*n* = 40)		*P* -value
	Mean (SD)	Median (range)	Mean (SD)	Median (range)	Mean/median
**CPQ_11–14_: total score**					
Total group	8.5 (5.6)	7.0 (20)	6.9 (5.6)	6.5 (28)	0.218/0.762
Boys	7.5 (4.3)	6.0 (18)	5.7 (4.0)	5.5 (17)	0.134/0.666
Girls	10.1 (6.9)	13.0 (20)	9.2 (7.4)	8.5 (28)	0.736/0.579
**Oral symptoms (OS)**					
Total group	3.5 (1.9)	4.0 (7)	2.9 (1.9)	3.0 (8)	0.148/0.081
Boys	3.7 (1.6)	4.0 (7)	2.8 (1.9)	3.0 (8)	0.098/0.170
Girls	3.2 (2.3)	4.0 (7)	2.9 (2.1)	3.0 (6)	0.743/0.436
**Functional limitations (FL)**					
Total group	1.3 (1.2)	1.0 (4)	1.9 (1.9)	2.0 (7)	0.087/0.495
Boys	1.1 (1.1)	1.0 (3)	1.8 (1.9)	1.0 (7)	0.133/0.646
Girls	1.6 (1.5)	2.0 (4)	2.2 (2.1)	2.0 (7)	0.371/0.659
**Emotional well-being (EW)**					
Total group	2.2 (2.4)	1.5 (8)	1.0 (2.0)	0.0 (11)	**0.018**/**0.044**
Boys	1.7 (2.0)	1.0 (5)	0.5 (0.9)	0.0 (3)	**0.006**/**0.034**
Girls	3.0 (3.0)	2.0 (8)	2 (3.0)	1.0 (11)	0.351/0.292
**Social well-being (SW)**					
Total group	1.5 (1.9)	1.0 (9)	1.1 (2.2)	0.0 (11)	0.458/**0.045**
Boys	1.0 (1.3)	1.0 (6)	1.2 (1.4)	0.0 (6)	0.326/**0.037**
Girls	2.2 (2.5)	2.0 (9)	2.0 (3.1)	1.0 (11)	0.854/0.369

Bold indicate *P* < 0.05.

The intervention group demonstrated significantly higher median scores in the domains of EW (*P* = 0.018) and SW (*P* = 0.045) than the control group. Furthermore, boys in the intervention group reported significantly higher mean and median scores in EW (*P* = 0.006 and *P* = 0.034, respectively) and higher median scores in SW (*P* = 0.037) compared with boys in the control group. No significant differences between groups were observed for OS or FL (Table 2).

Girls with higher total CPQ_11–14_ scores reported thinking more frequently about the residual gap (*P* = 0.034), whereas no such pattern was observed among boys (*P* = 0.927). No association was observed between total CPQ_11–14_ scores and interest in treatment for the remaining gap. Additionally, higher total CPQ_11–14_ scores were significantly associated with lower satisfaction with dental alignment among girls, but not among boys (*P* = 0.031 and *P* = 0.631, respectively). However, total scores were not related to the desire for orthodontic treatment in either girls or boys (*P* = 0.07 and *P* = 0.673).

The multiple linear regression analysis showed that the IOTN-DHC influenced significantly the total CPQ_11–14_ score while controlling for cofounders (*β* = 1.71; *P* = 0.002).

### Harms

No harmful effects were observed during this part of the study.

## Discussion

This study is the first to employ OHRQoL measures in patients who underwent hemisection and conventional extraction without subsequent orthodontic treatment, in comparison with a control group. In addition, patients’ recall of which interceptive procedure was perceived as more painful was assessed. The main findings showed that the mean total CPQ scores were similar between the groups and patients in the intervention group recalled significantly greater pain after hemisection compared with conventional extraction.

The low CPQ scores observed in the current study indicate that the participants generally experienced good OHRQoL. These findings are consistent with other Swedish studies [26, 27] but contrast with reports from other countries [16, 28]. OHRQoL may be influenced by cultural factors as well as differences in healthcare systems [16]. Several studies have demonstrated that malocclusion, particularly anterior malocclusion, negatively affects OHRQoL [15, 16, 28]. When treatment need is evaluated according to the IOTN–DHC, significant differences in OHRQoL have been reported; however, no clear association between lower OHRQoL and higher IOTN scores has been established [26]. In the present study, multiple regression analysis showed that the IOTN significantly influenced the total CPQ scores after controlling for confounders.

The impact of malocclusion on OHRQoL has primarily been reported within the domains of EW and SW [16, 26, 29, 30], suggesting that malocclusion may represent more of a psychosocial concern than an oral or functional problem [29, 31]. In the present study, although the total CPQ scores were similar between the groups, higher median scores for the EW and SW domains were observed in the intervention group. An unexpected finding was that boys in the intervention group reported significantly higher scores in EW and SW compared with boys in the control group, whereas girls in the intervention group with higher CPQ scores reported thinking more frequently about the residual gap. Nevertheless, total CPQ scores were not associated with the desire for orthodontic treatment in either girls or boys, and 82.5% of the patients did not wish to undergo treatment to close the remaining gaps. Previous literature generally reports that girls tend to be more dissatisfied with their dental appearance and more sensitive to health-related issues, while boys may underreport concerns, possibly due to social norms [26, 29, 32–34]. Children aged 11–14 years, who are undergoing substantial physical and psychosocial changes, may be particularly sensitive to the effects of malocclusions on OHRQoL [16, 35], which may explain why younger patients reports lower CPQ scores [27].

Several factors have been reported to influence OHRQoL. One such factor is caries, which has been shown to have twice as negative impact compared with dental trauma or malocclusion [36]. Headache is another contributing factor, particularly among girls, and mainly affects the OS domain [26, 27, 37]. In the present study, both groups showed a low prevalence of caries, and the majority did not report experiencing headaches, which may explain the generally low CPQ scores. The influence of dental fear on OHRQoL has also been documented [26, 38–40]. The development of dental fear is multifactorial and complex. It has been reported to be more prevalent among girls and to decrease with increasing age. Furthermore, dental fear is influenced by cultural beliefs, individual personality traits, and prior experiences, including both dental and medical encounters [41]. In this study, dental fear assessed using the CFSS-DS was recorded at only one time point in the intervention group, making it impossible to determine whether the extractions affected the scores. A comparison between the groups was therefore not feasible. Nevertheless, the low mean CFSS-DS scores in the intervention group suggest that none of the patients exhibited dental fear.

Other confounding factors reported to affect OHRQoL include dental pain, general health problems, previous hospitalization, and low socioeconomic status [38, 42]. These variables were not evaluated in the present study, with the exception of socioeconomic status; notably, none of the participants were identified as having a low socioeconomic level.

A noteworthy and clinically significant finding was that patients in the intervention group were able to recall, more than 4 years after treatment, that hemisection had been significantly more painful than conventional extraction. As this is the first study to evaluate OHRQoL in patients with congenitally missing second premolars who underwent interceptive extraction without subsequent orthodontic treatment, there are no previous studies available for direct comparison or validation of these results. At present, no specific instrument exists to assess how remaining gaps influence OHRQoL. Furthermore, spacing is not incorporated into the IOTN scoring system, which led to the inclusion of additional targeted questions in the questionnaire administered to the intervention group. The observation that most patients were not disturbed by the residual posterior gaps provides valuable insight into patient perspectives and contributes to a deeper understanding of how interceptive extraction of primary second molars affects patients in the long-term.

### Strengths and limitations

A major strength of this study is the comparison with a control group matched for age, sex, and geographical area. Furthermore, all examinations, semi-structured interviews, and questionnaires were conducted by a single operator, which increased consistency and reduced inter-examiner variability. The Swedish version of CPQ_11–14_ has demonstrated good validity and reliability and is recommended for assessing OHRQoL in Swedish children aged 11–14 years [22], which further strengthens the methodological quality of the study. However, OHRQoL was not assessed before and directly after the intervention, which precludes assessment of whether the treatment led to a measurable change over time. Recalling pain several years after treatment may also be challenging; nevertheless, the fact that a significant difference between hemisection and conventional extraction was detected strengthens the assumption that a true difference existed. In addition, CFSS-DS scores were not recorded prior to extraction, which limits the ability to draw conclusions about whether the interventions influenced patients’ dental fear. Furthermore, caries data were obtained from dental records and recorded by multiple clinicians without documented standardized training or calibration; therefore, the relevance of the caries data should be interpreted with caution.

Finally, the power calculation was based on differences in residual space between hemisection and extraction rather than on QoL outcomes. Consequently, the study may have been underpowered to detect differences in other outcome measures. In addition, 42 patients were enrolled in the intervention group, whereas 43 participants would have been required when accounting for the anticipated 30% dropout rate. However, since no patients withdrew after allocation to the intervention, the primary outcome used for the power calculation remained sufficiently powered.

### Generalizability

The relatively small sample of 80 patients limits the extent to which the findings can be generalized to a wider population. Patients’ acceptance of remaining gaps and OHRQoL may be influenced by personality traits as well as cultural, socioeconomical and social factors, and these perceptions may vary across countries.

### Clinical implications

Previous reports based on the intervention group have shown that extraction of mandibular primary second molars in patients with congenitally missing second premolars results in a mean residual space of 2.0 mm, accompanied by an average four-degree tipping of the permanent first molar and mesial space opening adjacent to the first permanent premolar. Hemisection was also associated with more postoperative symptoms and higher costs [8]. The present study further supports these patient-reported findings, as participants were able to recall, 4 years after the intervention, experiencing significantly more pain following hemisection compared with conventional extraction.

The remaining gaps were not closed with additional treatment, as small posterior spaces that do not result in functional or esthetic impairment are not prioritized for orthodontic care in Sweden. The Swedish dental care system is publicly funded up to the age of 19, and only patients who meet specific criteria are eligible for cost-free orthodontic treatment. Therefore, the findings of this study are of considerable importance, as more than 80% of patients in the intervention group were not interested in receiving further treatment to close the residual spaces and there were no differences in the total CPQ scores between the intervention and the control group.

## Conclusion

- Hemisection was recalled as more painful than extraction.
- The majority of the patients did not express a desire for closure of the residual gaps.
- No significant differences were found in overall oral health–related quality of life between the groups, indicating that small posterior gaps did not adversely affect patients’ long-term perception of oral health.

## Data Availability

All data generated or analysed during this study are included in this article. Further enquiries can be directed to the corresponding author.

## References

[1] Fekonja A . Hypodontia in orthodontically treated children. Eur J Orthod 2005;27:457–60. 10.1093/ejo/cji027 16043466

[2] Thilander B, Myrberg N. The prevalence of malocclusion in Swedish schoolchildren. Scand J Dent Res 1973;81:12–21. 10.1111/j.1600-0722.1973.tb01489.x 4510864

[3] Ravn JJ . Aplasia, supernumerary teeth and fused teeth in the primary dentition. An epidemiologic study. Scand J Dent Res 1971;79:1–6. 10.1111/j.1600-0722.1971.tb01986.x 5292961

[4] Grahnén H . Hypodontia in the Permanent Dentition, a Clinical and Genetical Investigation. Lund: Gleerup, 1956. Print.

[5] Bozga A, Stanciu RP, Mănuc D. A study of prevalence and distribution of tooth agenesis. J Med Life 2014;7:551–4. 25713620 PMC4316137

[6] Polder BJ, Van't Hof MA, Van der Linden FP et al A meta-analysis of the prevalence of dental agenesis of permanent teeth. Community Dent Oral Epidemiol 2004;32:217–26. 10.1111/j.1600-0528.2004.00158.x 15151692

[7] Bjerklin K, Bennett J. The long-term survival of lower second primary molars in subjects with agenesis of the premolars. Eur J Orthod 2000;22:245–55. 10.1093/ejo/22.3.245 10920557

[8] Abdul Jabbar S, Nawaia S, Rughwani V et al Hemisection versus conventional extraction as interceptive treatment in congenitally missing mandibular second premolars: a randomised controlled split-mouth trial. Eur J Orthod 2025;47:cjaf043. 10.1093/ejo/cjaf043 40501277 PMC12159412

[9] Josefsson E, Brattström V, Tegsjö U et al Treatment of lower second premolar agenesis by autotransplantation: four-year evaluation of eighty patients. Acta Odontol Scand 1999;57:111–5. 10.1080/000163599429002 10445365

[10] Kokich VG, VO K. Congenitally missing mandibular second premolars: clinical options. Am J Orthod Dentofacial Orthop 2006;130:437–44. 10.1016/j.ajodo.2006.05.025 17045142

[11] Williams R, Park JH, Chae JM et al The congenitally missing second premolar: space closure. A viable option. Am J Orthod Dentofacial Orthop 2020;157:571–583.e16. 10.1016/j.ajodo.2019.10.015 32241364

[12] Locker D, Allen F. What do measures of ‘oral health-related quality of life’ measure? Community Dent Oral Epidemiol 2007;35:401–11. 10.1111/j.1600-0528.2007.00418.x 18039281

[13] Kressin NR . The oral health related quality of LifeMeasure (OHQOL). In: Slade GD, (ed.) Measuringoral Health and Quality of Life. Chapel Hill: Universityof North Carolina, Dental Ecology, 1997, 114–9.

[14] Barbosa TS, Gaviao MB. Oral health-related quality of lifein children: part I. How well do children know themselves?A systematic review. Int J Dent Hyg 2008;6:93–9. 10.1111/j.1601-5037.2007.00276.x 18412720

[15] Dimberg L, Arnrup K, Bondemark L. The impact of malocclusion on the quality of life among children and adolescents: a systematic review of quantitative studies. Eur J Orthod 2015;37:238–47. 10.1093/ejo/cju046 25214504

[16] Kragt L, Dhamo B, Wolvius EB et al The impact of malocclusions on oral health-related quality of life in childrena systematic review and meta-analysis. Clin Oral Investig 2016;20:1881–94. 10.1007/s00784-015-1681-3 PMC5069349 26635095

[17] Jena AK, Mohapatra M, Sharan J et al Temporary deterioration of oral health-related quality of life (OHRQoL) in nonextraction and extraction modalities of comprehensive orthodontic treatment in adolescents. Angle Orthod 2020;90:578–86. 10.2319/092319-607.1 33378501 PMC8028460

[18] DiBiase A, Cox Z, Rea M et al Malocclusion and peer relationships in school children aged 10–14 years in the United Kingdom: a cross-sectional study. Am J Orthod Dentofacial Orthop 2025;168:435–450. 10.1016/j.ajodo.2025.04.017 40407766

[19] Kotecha S, Turner PJ, Dietrich T et al The impact of tooth agenesis on oral health-related quality of life in children. J Orthod 2013;40:122–9. 10.1179/1465313312Y.0000000035 23794692

[20] Liu Z, McGrath C, Hagg U. The impact of malocclusion/orthodontic treatment need on the quality of life. A systematic review. Angle Orthod 2009;79:585–91. 10.2319/042108-224.1 19413386

[21] Nawaia S, Al-Taai N, Abdul Jabbar S et al Dentoalveolar changes following extraction of mandibular primary second molars in patients with congenitally missing second premolars-a longitudinal randomized controlled trial. Eur J Orthod 2025;47:cjaf095. 10.1093/ejo/cjaf095 41258792 PMC12628498

[22] Dimberg L, Lennartsson B, Bondemark L et al Validity and reliability of the Swedish versions of the short-form child perceptions questionnaire 11–14 and parental perceptions questionnaire. Acta Odontol Scand 2019;77:630–5. 10.1080/00016357.2019.1634282 31267808

[23] Jokovic A, Locker D, Guyatt G. Short forms of the child perceptions questionnaire for 11–14-year-old children (CPQ_11–14_): development and initial evaluation. Health Qual Life Outcomes 2006;4:4. 10.1186/1477-7525-4-4 16423298 PMC1368964

[24] Brook PH, Shaw WC. The development of an index of orthodontic treatment priority. Eur J Orthod 1989;11:309–20. 10.1093/oxfordjournals.ejo.a035999 2792220

[25] Klingberg G . Reliability and validity of the Swedish version of the dental subscale of the children’s fear survey schedule, CFSS-DS. Acta Odontol Scand 1994;52:255–6. 10.3109/00016359409029055 7985512

[26] Dimberg L, Lennartsson B, Bondemark L et al Oral health-related quality-of-life among children in Swedish dental care: the impact from malocclusions or orthodontic treatment need. Acta Odontol Scand 2016;74:127–33. 10.3109/00016357.2015.1059485 26206412

[27] Kallunki J, Sollenius O, Paulsson L et al Oral health-related quality of life among children with excessive overjet or unilateral posterior crossbite with functional shift compared to children with no or mild orthodontic treatment need. Eur J Orthod 2019;41:111–6. 10.1093/ejo/cjy033 29878165

[28] Simões RC, Goettems ML, Schuch HS et al Impact of malocclusion on oral health-related quality of life of 8–12 years old schoolchildren in southern Brazil. Braz Dent J 2017;28:105–12. 10.1590/0103-6440201701278 28301027

[29] Scapini A, Feldens CA, Ardenghi TM et al Malocclusion impacts adolescents’ oral health-related quality of life. Angle Orthod 2013;83:512–8. 10.2319/062012-509.1 23210545 PMC8763080

[30] Agou S, Locker D, Muirhead V et al Does psychological well-being influence oral-health-related quality of life reports in children receiving orthodontic treatment? Am J Orthod Dentofacial Orthop 2011;139:369–77. 10.1016/j.ajodo.2009.05.034 21392693

[31] Krekmanova L, Shakrchi S, Gicic A et al Comparison of the opinions of adolescents with different orthodontic treatment needs. Clin Exp Dent Res 2024;10:e944. 10.1002/cre2.944 39205456 PMC11358389

[32] Deli R, Macrì LA, Radico P et al Orthodontic treatment attitude versus orthodontic treatment need: differences by gender, age, socioeconomical Status and geographical context. Supplement, Community Dent Oral Epidemiol 2012;40:71–6. 10.1111/j.1600-0528.2011.00669.x 22369712

[33] Christopherson EA, Briskie D, Inglehart MR. Objective, subjective, and self-assessment of preadolescent orthodontic treatment need—A function of age, gender, and ethnic/racial background? J Public Health Dent 2009;69:9–17. 10.1111/j.1752-7325.2008.00089.x 18662255

[34] Mc Grath C, Bedi R. Gender variations in the social impact of oral health. J Ir Dent Assoc 2000;46:87–91. 11323941

[35] Meade T, Dowswell E. Adolescents’ health-related quality of life (HRQoL) changes over time: a three year longitudinal study. Health Qual Life Outcomes 2016;14:14. 10.1186/s12955-016-0415-9 26810328 PMC4727407

[36] Martins MT, Sardenberg F, Bendo CB et al Dental caries remains as the main oral condition with the greatest impact on children’s quality of life. PLoS One 2017;12:e0185365. 10.1371/journal.pone.0185365 28981545 PMC5628830

[37] Albers L, Von Kries R, Heinen F et al Headache in school-children: prevalence increasing. Curr Pain Headache Rep 2015;19:477. 10.1007/s11916-015-0477-0 25754597

[38] Merdad L, El-Housseiny AA. Do children’s previous dental experience and fear affect their perceived oral health-related quality of life (OHRQoL)? BMC Oral Health 2017;17:47. 10.1186/s12903-017-0338-9 28093086 PMC5240375

[39] Luoto A, Lahti S, Nevanpera T et al Oral-health-related quality of life among children with and without dental fear. Int J Paediatr Dent 2009;19:115–20. 10.1111/j.1365-263X.2008.00943.x 19250394

[40] Carillo-Diaz M, Crego A, Romero-Maroto M. The influence of gender on relationship between dental anxiety and oralhealth- related emotional well-being. Int J Paediatr Dent 2013;3:180–7. 10.1111/j.1365-263X.2012.01242.x 22594301

[41] Cianetti S, Lombardo G, Lupatelli E et al Dental fear/anxiety among children and adolescents. A systematic review. Eur J Paediatr Dent 2017;18:121–30. 10.23804/ejpd.2017.18.02.07 28598183

[42] Schuch HS, Correa MB, Torriani DD et al Perceived dental pain: determinants and impact on Brazilian schoolchildren. J Oral Facial Pain Headache 2015;29:168–76. 10.11607/ofph.1414 25905535

